# Evaluation of Serum Calprotectin as an Alternative Diagnostic Marker for Intrahepatic Cholestasis of Pregnancy

**DOI:** 10.3390/jcm13185644

**Published:** 2024-09-23

**Authors:** Harshita Katiyar, Sangeeta Yadav, Surender Singh, Ajay Kumar Mishra, Mandakini Pradhan, Raghavendra Lingaiah, Amit Goel

**Affiliations:** 1 Department of Hepatology, Sanjay Gandhi Postgraduate Institute of Medical Sciences, Lucknow 226014, India; harshita9katiyar@gmail.com (H.K.); surmmcian2k6@gmail.com (S.S.); ajaymishrapandit@gmail.com (A.K.M.); 2 Department of Maternal & Reproductive Health, Sanjay Gandhi Postgraduate Institute of Medical Sciences, Lucknow 226014, India; sangeetasgpgi@gmail.com (S.Y.); mpradhan9918@gmail.com (M.P.); 3 Department of Pathology, Sanjay Gandhi Postgraduate Institute of Medical Sciences, Lucknow 226014, India; raghulingaiah@gmail.com

**Keywords:** cholestasis, pregnancy, conjugated hyperbilirubinemia, jaundice, bile acid

## Abstract

**Background/Objectives**: Intrahepatic cholestasis of pregnancy (ICP) is characterised by unexplained intense pruritus during pregnancy. While serum bile acid (BA) is the standard diagnostic marker for ICP, we explored the potential of serum calprotectin as an alternative diagnostic marker for ICP. **Methods**: Leftover serum specimens with known serum BA levels, collected from non-pregnant women and pregnant women with an ICP, were used to measure serum calprotectin levels using the Human calprotectin L1/S100-A8/A9 ELISA kit. **Results**: Serum calprotectin levels were measured in 79 pregnant women with ICP (median [interquartile range] 28 year; serum BA 20 [13.7–35.7] μMol/L; calprotectin159 pg/mL [122.2–212.3]); 43 pregnant women without ICP (age 28 years; serum BA 3.6 [2.1–5.8] μMol/L; calprotectin 146.5 pg/mL [75.8–194.8]), and 59 non-pregnant women (age 28 years; serum BA 3.5 [1.6–5.1 μMol/L; calprotectin 82.4 pg/mL [48.8–137.2]). Compared to non-pregnant women, calprotectin levels were significantly elevated among pregnant women with (*p* < 0.001) or without ICP (*p* = 0.01). Calprotectin levels were comparable between pregnant women with and without ICP (*p* = 0.15). The areas under the ROC curve, to differentiate the presence and absence of ICP, were 0.940 (0.903–0.977; *p* < 0.001) and 0.681 (0.604–0.759; *p* < 0.001) for BA and calprotectin, respectively. **Conclusions**: Serum calprotectin is raised in pregnant women regardless of the presence or absence of ICP and had an inferior diagnostic performance for ICP compared to BA. This information is crucial for understanding the challenges in ICP diagnosis and the limitations of serum calprotectin as an alternative marker.

## 1. Introduction

Intrahepatic cholestasis of pregnancy (ICP) is a common liver disease in pregnant women [1]. The prevalence of ICP varies widely globally [2], and it is relatively lower in European countries [3] than in Scandinavian countries and Chile [4]. In a study from India, the prevalence was 2.8% among pregnant women attending maternal health care services [5].

ICP is characterised by unexplained intense pruritus, particularly in the palms and soles at night, during the third trimester of gestation. A proportion of affected women may also have associated mild elevation of liver enzymes and serum bilirubin [6]. ICP is a benign condition with favourable outcomes for the mother. Still, it may adversely affect the foetal outcome in terms of preterm delivery, meconium staining of amniotic fluid, foetal bradycardia, foetal distress, and foetal death [7,8,9]. All these risks are higher with higher bile acid (BA) levels in maternal serum. Hence, it is of the utmost importance to early identify antenatal women with ICP so that necessary arrangements can be made to manage a high-risk pregnancy and high-risk baby at birth. The diagnosis of ICP relies on typical clinical features in combination with elevated serum BA levels. Most of the significant international obstetrics and gynaecology guidelines recommend the measurement of serum BA to diagnose ICP, and a serum level > 10 μmol/L is recommended as a diagnostic marker for ICP [10].

Serum BA measurement has been universally accepted as a diagnostic serum marker for the diagnosis of ICP. BA measurement has certain limitations, such as the serum level being influenced by the diet [11], limited availability of its measurement, and the relatively lower sensitivity and specificity that are needed to diagnose ICP [12]. In a recent study, a BA level cut-off of >10 μmol/L had ~85% sensitivity in diagnosing ICP [5]. To overcome these limitations, researchers are constantly searching for new diagnostic markers for ICP. Several novel maternal serum markers, such as long noncoding RNA (lncRNAs) [13] and YKL-40 [14], have been evaluated to diagnose ICP. Still, they were found to have limited performance compared to serum BA.

Serum calprotectin has recently been evaluated as a diagnostic marker for ICP [15]. This case–control study was conducted in the Turkish population and measured serum calprotectin levels in 45 women with ICP (cases) and 45 pregnant women without ICP (control group). Serum calprotectin levels were markedly elevated among ICP (765.4 ± 126.8 [515.0–931.5] μg) compared to controls (48.0 ±10.4 [31.6–75.2] μg). The study mentioned above had a few limitations, such as not comparing the serum calprotectin levels of pregnant females with those of non-pregnant ones. Further, the data from this study need to be validated in other populations before they can be used for clinical use in different countries. The present study aims to measure the serum calprotectin level as a marker of ICP and compare its performance with serum BA to diagnose ICP.

## 2. Materials and Methods

### 2.1. Study Population

This cross-sectional study was conducted between January 2022 and December 2022 in the Department of Maternal and Child Health, Sanjay Gandhi Postgraduate Institute of Medical Sciences, Lucknow, India. Our recent work estimated serum bile acid levels in women with intrahepatic cholestasis of pregnancy compared to healthy non-pregnant and pregnant women [5]. After written informed consent was obtained, a blood sample was taken to estimate the bile acid level. The blood specimens used for calprotectin measurement in the present study were the leftover specimens collected from prospectively enrolled women in our previous study. That prospective study was conducted in our institution’s Department of Maternal and Reproductive Health between February 2017 and January 2019. All the leftover specimens were then used for calprotectin estimation. Our institute’s ethics committee also approved the previous study. In this study, we excluded the women who had another cause of liver injury, e.g., symptomatic gallstone disease, positive serology for hepatitis B or C, hepatotoxic drug intake, history of any disease of the hepatobiliary system, any systemic or metabolic disease with hepatic manifestations, or any chronic inflammatory diseases like Chron’s disease, ulcerative colitis, rheumatoid arthritis, systemic lupus erythematosus, vasculitis, or any active infection.

### 2.2. Serum Bile Acid Measurement

Serum BA levels were assessed by an enzyme-based and commercially available colourimetric assay. (Total Bile Acid, 5th generation colorimetric Rx Assay; Randox Laboratories, Crumlin, UK). An enzymatic reaction converted the serum BA and thionicotinamide adenine dinucleotide in the assay reagent to 3-ketosteroids and the reduced form of thioNAD. The rate of formation of the reduced form of thio-NADH was determined by measuring the change in absorbance at 405 nm. The lower detection limit for bile acid was 3.20 mmol/L, and the highest reading was 188 mmol/L.

### 2.3. Serum Calprotectin Measurement

Serum calprotectin was measured using a Human calprotectin L1/S100-A8/A9 ELISA kit (Thermo-Fisher Scientific, Waltham, MA, USA) as per the standard protocol recommended by the manufacturer. The Human Calprotectin L1/S100-A8/A9 Complex ELISA Kit is a solid-phase sandwich Enzyme-Linked Immunosorbent Assay (ELISA) designed to detect and quantify the level of human Calprotectin L1 in cell culture supernatants, plasma, and serum. The analytical sensitivity of Calprotectin L1 was 35 pg/mL, with an assay range of 35 pg/mL to 8000 pg/mL. The assay exclusively recognised natural and recombinant human Calprotectin L1/S100-A8/A9 Complexes. The S100A8/A9 heterocomplex is also called the MRP-8/MRP-14 complex or calprotectin.

### 2.4. Statistical Analysis

Categorical and continuous variables were expressed as ratio/proportions and median (interquartile range). Intergroup comparison was done using Chi2 and Wilcoxon’s rank-sum test and STATA software (Stata Statistical Software: Release 16; version 16.1, StataCorp LLC, College Station, TX, USA). The diagnostic performance of the serum BA and serum calprotectin was assessed with the receiver operating curve (ROC). *p* value < 0.05 was considered significant.

## 3. Results

Serum calprotectin was measured in the leftover serum specimens of 79 pregnant women with ICP (28 [16,17,18,19,20] years; BMI 26.5 [24.7–28.9] Kg/m^2^; serum BA 20 [13.7–35.7] μMol/L); 43 pregnant women without ICP (age 28 [16,17,18,19,20,21] years; 24.7 [23.4–26.6] Kg/m^2^; serum BA 3.6 [2.1–5.8] μMol/L) and 59 non-pregnant women (age 28 [16,17,18,19,20,21] years; BMI 23.4 [22.0–24.8] Kg/m^2^; serum BA 3.5 [1.6–5.1 μMol/L]), and data were included in the analysis (Table 1 and Table 2).

**Figure 1:**
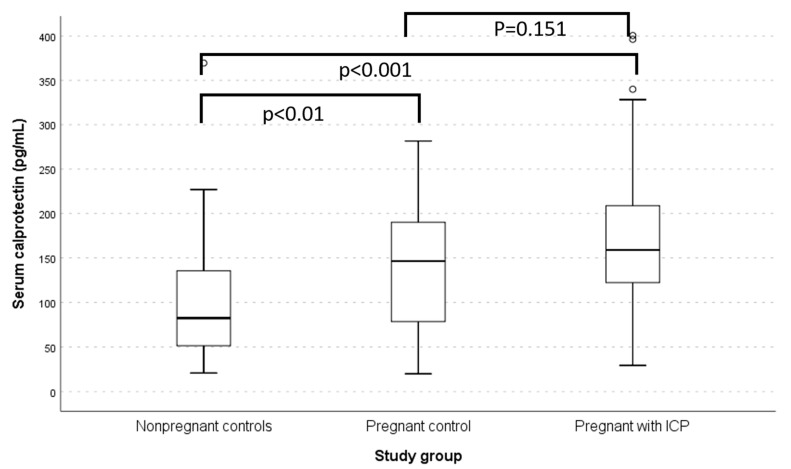
Box plot showing the difference in the serum calprotectin levels between the non-pregnant controls, pregnant women without ICP, and pregnant women with ICP.

On comparing the markers between pregnant women with and without ICP, serum ALT, AST, and bile acid were significantly elevated in the presence of ICP. Still, their serum calprotectin was comparable (Figure 1). As compared to non-pregnant women, serum calprotectin levels were significantly elevated among pregnant women with (*p* < 0.001) and without ICP (*p* = 0.01) (Table 2).

**Figure 2:**
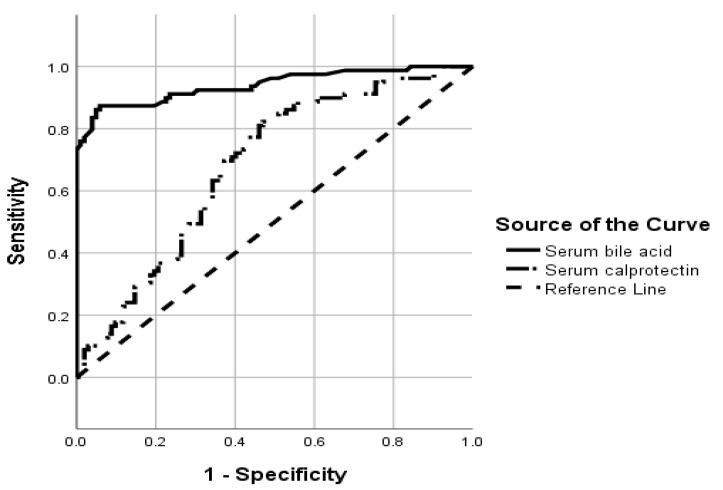
The receiver operating characteristic (ROC) curve shows the area under the curve (AUC) of serum bile acids and serum calprotectin when diagnosing intrahepatic cholestasis of pregnancy.

The area under the curve (AUC) of the receiver operating characteristic (ROC) curve, to differentiate the presence and absence of ICP, was 0.940 (0.903–0.977; *p* < 0.001) for BA and 0.681 (0.604–0.759; *p* < 0.001) for calprotectin, respectively (Figure 2).

Among pregnant women with ICP, the serum levels of BA and calprotectin were comparable between the groups with different levels of serum ALT elevations (Table 3); further, serum calprotectin levels correlated very poorly with serum levels of BA (correlation coefficient r = −0.091), ALT (r = 0.125), AST (r = 0.073), and maternal gestational age (r = 0.017). Serum calprotectin levels were comparable between the ICP women with serum BA < 10 μMol/L (166.4 [139.8–250.9]), 10–19.9 μMol/L (156.4 [127.3–212.9]), 20–29.9 μMol/L (157.5 [92.4–206.5]), 30–39.9 μMol/L (164.2 [119–192.8]), and ≥40 μMol/L (157.2 [115.2–199.9]).

## 4. Discussion

In this cross-sectional study, we compared serum calprotectin levels between pregnant women with ICP, pregnant women without ICP, and non-pregnant women. Calprotectin levels were significantly higher in pregnant women than in non-pregnant women, regardless of their ICP status. The presence of ICP in pregnant women was associated with higher serum levels of ALT, AST, and BA. Serum calprotectin levels in pregnant women were not affected by the presence of ICP. In our cohort, BA had a much better diagnostic performance for ICP than calprotectin.

ICP is an enigmatic condition of pregnancy, and its pathogenesis remains unexplained. Family history of ICP, gestational diabetes, multiple gestation, high-dose oral contraceptives, hormonal treatment during pregnancy [3], and preeclampsia are the risk factors for ICP [22]. The role of environmental factors, gestational hormones, hereditary factors [23], and mutations of the hepatobiliary transport proteins has been extensively explored in the pathogenesis of ICP [24] without any conclusion. In recent years, immune-mediated injury has also been studied in the pathogenesis of ICP [25].

Inflammatory cell activation and induction of proinflammatory cytokines have also been recognised as causes of ICP. Bile formation and secretion swiftly reduce as an adaptation to infection and inflammation. Cholestasis is a negative acute phase reaction of the liver to lipopolysaccharide (LPS) or endotoxemia-medicated inflammation [26]. Circulation LPS induces Kupffer cells to locally produce high levels of proinflammatory cytokines, activating the membrane receptors of hepatocytes and cholangiocytes, transducing intracellular signals, and culminating in altered expression and function of transporter and excretory proteins—these result in impaired bile formation and excretion, i.e., intrahepatic cholestasis [26].

Several circulating markers of inflammation are examined in diagnosing ICP. Interleukin-6 (IL-6) and high-sensitivity CRP (hs-CRP) are elevated in those with ICP compared to those in control groups [27]. Recently, serum calprotectin, another marker of inflammation, has been found to be markedly elevated in women with ICP compared to healthy controls [15].

Calprotectin, a member of the S100 protein family, is a calcium- and zinc-binding cytoplasmic protein present in neutrophils; in addition, it is also found, though in a smaller quantity, in monocytes, macrophages, epithelial cells, and endothelial cells. Its expression is mainly induced at the time of cell activation. Calprotectin, released via a novel tubulin-dependent mechanism, controls the intracellular pathways of innate immune cells and the inflammatory response [28]. Calprotectin has been widely used as a marker of inflammation for various conditions, such as inflammatory bowel disease, rheumatoid arthritis, psoriasis, cancers, and neurodegenerative disease [29]. Such widespread use of calprotectin reflects its features, which favour its use as a disease marker, such as its stability, high diagnostic sensitivity, reproducibility, and low cost.

Our data revealed two critical observations. First, calprotectin levels were elevated in pregnant women without ICP as compared to non-pregnant controls. Calprotectin and other inflammatory serum markers can be elevated in pregnant women with pregnancy-related complications other than ICP or infections, such as preeclampsia [16,21,30], other hypertensive disorders [17], and diabetes mellitus [18]. Pregnancy is a state characterised by an intense disharmony of maternal endocrine and immune systems required to sustain the pregnancy. This leads to systemic low-grade inflammation and may result in the elevation of maternal serum calprotectin levels. Another recent study has shown that faecal calprotectin gradually increases in healthy women as the pregnancy advances, supporting our finding of elevated serum calprotectin in pregnant controls [19].

Second, we found that calprotectin levels were comparable between pregnant women with and without ICP; calprotectin levels did not vary with serum bile acid levels. This contrasts with a previous study, which showed a significantly higher calprotectin level in the ICP group [15]. The presence of ICP is known to be associated with poor foetal outcomes [8]. One of the mechanisms for adverse foetal outcomes is bile acid-mediated placental insufficiency, leading to foetal hypoxia [20]. The effect of bile acid on the placenta may induce calprotectin release from the affected endothelial lining, resulting in a rise in maternal calprotectin levels. We need more data to explain the exact pathogenesis of the increase in serum calprotectin and reach a conclusion.

Our study’s strengths include a relatively reasonable sample size and the inclusion of pregnant and non-pregnant controls. We had not collected data on preeclampsia or pregnancy-induced hypertension, which could have affected the calprotectin level in control groups.

Our data also have a few limitations. First, preeclampsia may result in elevated calprotectin levels in serum, and this information is missing from our study participants. Second, we used stored serum samples for calprotectin level estimation, which could have led to an underestimation of their level due to their natural disintegration over time. Calprotectin is an inflammatory marker, but we had not measured any other marker of inflammation, such as C reactive protein, in serum samples.

## 5. Conclusions

Serum calprotectin is raised in pregnant women, regardless of the presence or absence of ICP. It correlates very poorly with serum bile acid levels. Compared to serum bile acid, serum calprotectin has an inferior diagnostic performance for ICP and is unlikely to be an alternative for bile acid measurement to diagnose ICP.

## Figures and Tables

**Table 1:** Comparison of the baseline clinical and laboratory parameters of pregnant women with and without intrahepatic cholestasis of pregnancy.

Characteristics	Healthy Pregnant Women (*n* = 43)	Pregnant Women with ICP (*n* = 79)	Non-Pregnant Control (*n* = 59)	*p* Value
Age (years)	28 (25–31)	28 (26–32)	28 (25–32)	0.914
BMI (Kg/m^2^)	24.7 (23.4–26.6)	26.5 (24.7–28.9	23.4 (22.0–24.8)	<0.001
Primigravida	10	26	Not applicable	
Gestational age (weeks)	35 (33–37)	33 (30–36)	Not applicable	0.074
Associated conditions			Not applicable	
Hypothyroidism	6	14		
Gestational diabetes	10	3		
Liver function test			Not applicable	
Total serum bilirubin	0.6 (0.5–0.7)	0.7 (0.6–0.9)		0.017
ALT (IU/L)	24 (18–34)	142 (98–285)		<0.001
AST (IU/L)	23 (16–34)	118 (78–232)		<0.001

BMI, body mass index; data are expressed as number (%) or median (interquartile range).

**Table 2:** Comparison of Serum markers among study groups.

Serum Marker	Non-Pregnant Women (*n* = 59)	Pregnant Women without ICP (*n* = 43)	*p* Value for Non-Pregnant Women and Pregnant Women without ICP	Pregnant Women with ICP (*n* = 79)	*p* Value for Pregnant Women with or without ICP
Serum bile acids (μMol/L)	3.5 (1.6–5.1)	3.6 (2.1–5.8)	0.49	20.0 (13.7–35.7)	<0.01
Serum calprotectin (pg/mL)	82.4 (48.8–137.2)	146.5 (75.8–194.8)	0.01	159.0 (122.2–212.3)	0.15

ICP, intrahepatic cholestasis of pregnancy; data are expressed as median (interquartile range) and compared using non-parametric tests.

**Table 3:** Serum bile acids and calprotectin levels in pregnant women with intrahepatic cholestasis with different levels of alanine aminotransferase elevation.

Serum Marker	ALT 41–100 IU/L (*n* = 23)	ALT 101–200 IU/L (*n* = 27)	ALT 201–400 IU/L (*n* = 19)	ALT > 400 IU/L (*n* = 10)	*p* Value
Serum bile acid (μMol/L)	14.1 (4.1–28.8)	20.0 (15.7–31.7)	25.0 (15.1–52.8)	29.9 (13.3–72.3)	0.056
Serum calprotectin (pg/mL)	144.1 (116–212.3)	166.4 (129–205)	164.5 (115.2–217.8)	173.1 (141.7–242.7)	0.663

Data are expressed as median (interquartile range).

## Data Availability

All the data supporting this study’s findings are available from the corresponding author upon reasonable request.
